# From Genes to Malformations: Molecular Mechanisms Driving the Pathogenesis of Congenital Anomalies of the Kidney and Urinary Tract

**DOI:** 10.3390/ijms27010017

**Published:** 2025-12-19

**Authors:** Maria Fourikou, John Dotis

**Affiliations:** Third Department of Pediatrics, Aristotle University of Thessaloniki, Hippokration Hospital, 54642 Thessaloniki, Greece; mfour@auth.gr

**Keywords:** congenital anomalies of the kidney and urinary tract, kidney development, monogenic variants, copy number variations, epigenetics, environmental exposure

## Abstract

Congenital Anomalies of the Kidney and Urinary Tract (CAKUT) are among the most common congenital malformations and the leading cause of chronic kidney disease in children. They arise when key steps in kidney development are disrupted, including ureteric bud induction, branching morphogenesis and nephron progenitor differentiation. These processes depend on coordinated transcriptional programs, signaling pathways, ciliary function and proper extracellular matrix (ECM) organization. Advances in whole exome and whole genome sequencing, as well as copy number variation analysis, have expanded the spectrum of known monogenic causes. Pathogenic variants have now been identified in major transcriptional regulators and multiple ciliopathy-related genes. Evidence also points to defects in central signaling pathways and changes in ECM composition as contributors to CAKUT pathogenesis. Clinical presentations vary widely, shaped by modifying effects of genetic background, epigenetic regulation and environmental influences such as maternal diabetes and fetal hypoxia. Emerging tools, including human kidney organoids, gene-editing approaches and single-cell or spatial transcriptomics, allow detailed exploration of developmental mechanisms and validation of candidate pathways. Overall, CAKUT reflects a multifactorial condition shaped by interacting genetic, epigenetic and environmental determinants. Integrating genomic data with experimental models is essential for improving diagnosis, deepening biological insight and supporting the development of targeted therapeutic strategies.

## 1. Introduction

Congenital anomalies of the kidney and urinary tract (CAKUT) are the most frequent structural malformations detected in newborns and a leading cause of chronic kidney disease (CKD) in childhood [1]. The term includes a broad range of phenotypes, from mild or subclinical presentations such as isolated vesicoureteral reflux (VUR) to severe, life-threatening conditions including bilateral renal agenesis, obstructive uropathies and multicystic dysplastic kidneys [2,3]. Disruptions in nephrogenesis, ureteric bud (UB) development or urinary tract morphogenesis give rise to these defects, all relying on tightly regulated molecular and cellular mechanisms shaped by major signaling pathways and transcriptional networks [4,5,6].

Recent advances in genomic sequencing technologies, including whole-exome sequencing (WES), whole-genome sequencing (WGS) and copy-number variant (CNV) analysis, have broadened the list of genes linked to CAKUT. Although CNVs were traditionally considered a distinct analytical category, they can be detected using both WES- and WGS-based platforms, with WGS providing more comprehensive coverage of noncoding regions and improved sensitivity for complex or nonrecurrent structural variants, whereas WES-based CNV detection is largely restricted to coding regions. Emerging third-generation long-read sequencing technologies further enhance structural variant resolution by enabling accurate characterization of breakpoint architecture and repeat-rich regions, which are increasingly recognized as contributors to CAKUT pathogenesis [7].

Implicated loci range from transcriptional regulators, such as hepatocyte nuclear factor 1-beta (*HNF1B*), forkhead box D2 (*FOXD2*) and teashirt zinc finger homeobox 3 (*TSHZ3*), to ciliary and centrosomal proteins (CEPs), developmental signaling mediators and ribonucleic acid (RNA) processing factors [8,9,10,11,12]. Despite these advances, the genetic cause remains unidentified in most affected individuals and large cohorts consistently show that monogenic variants explain only a minority of cases. This gap highlights the contribution of noncoding variants, epigenetic mechanisms and multifactorial gene–environment interactions. Recent exome sequencing studies have revealed extensive genetic heterogeneity in CAKUT and frequent overlap with genes involved in broader developmental disorders [13]. Clinical sequencing datasets reporting diagnostic yields below 30% further underscore how many genetic contributors remain undiscovered [14].

Severity varies widely, even among individuals with the same pathogenic variant, indicating roles for genetic modifiers, epigenetic influences and environmental factors [1,6,15]. Nutritional status, maternal metabolic disorders and alterations in retinoic acid or vitamin D signaling may modulate developmental pathways and shape the degree or laterality of renal malformations [16]. Findings from CNV studies, including variants that affect miRNA loci, further underscore the importance of regulatory elements, rather than coding sequence alone, in determining CAKUT phenotypes [17,18]. Emerging genetic analyses also suggest oligogenic or polygenic contributions in a subset of patients, reflecting the complexity of renal developmental pathways [13].

Experimental models have become essential for dissecting CAKUT biology. Studies in animal systems, human kidney organoids and targeted molecular assays have identified abnormalities in ciliogenesis, extracellular matrix (ECM) organization, nephron patterning and transcriptional regulation [6,19,20,21]. These findings also show that many CAKUT-associated genes have pleiotropic developmental roles, helping explain the frequent involvement of other organ systems [13,21]. Importantly, although limited in availability, studies using human embryonic kidney tissue provide direct evidence that several CAKUT candidate genes and their corresponding proteins are expressed during normal human nephrogenesis, thereby strengthening the biological relevance of findings derived from experimental and in vitro models [22].

Improved understanding of the molecular basis of CAKUT is beginning to influence clinical practice. Genotype–phenotype correlations, such as the links between *HNF1B* and metabolic disorders [11], *FOXD2* and craniofacial anomalies [10] or *TSHZ3* and neurodevelopmental features [8], support more focused diagnostic evaluation and inform prognostic discussions. The recognition of CAKUT within multisystem developmental syndromes further emphasizes the value of comprehensive genetic testing in appropriate cases [14,23,24].

In this review, we summarize current knowledge on the developmental and molecular foundations of CAKUT. We integrate findings from human genetics, experimental modeling and genomic approaches to outline key pathways in kidney and urinary tract development. We also discuss the roles of CNVs, monogenic variants, epigenetic influences and environmental factors, as well as consider how emerging tools may reshape future diagnostic and therapeutic strategies.

## 2. Embryologic and Molecular Foundations of Kidney Development

Kidney formation relies on reciprocal signaling between the UB and the metanephric mesenchyme (MM), two progenitor tissues that regulate bud initiation, branching architecture, nephron formation and the organization of the lower urinary tract. Any interruption in this carefully coordinated developmental program can lead to a wide spectrum of CAKUT [4,5,6,15,25,26].

### 2.1. Specification and Initiation of the Metanephric Anlage

The process begins with signals from the MM, primarily involving the secretion of glial cell line-derived neurotrophic factor (*GDNF*). *GDNF* activates the rearranged during transfection (*RET*) receptor, together with its co-receptor glial cell line-derived neurotrophic factor family receptor alpha-1 (GFRα1), expressed on the Wolffian duct epithelium. This interaction initiates the emergence of the UB formation [10,11,12,14,16,27,28,29].

Disruption of this signaling axis may result in renal agenesis or dysplastic development of the kidney [29,30,31]. Several transcription factors, such as Wilms tumor protein 1 (*WT1*), sine oculis homeobox homolog 2 (*SIX2*), eyes absent homolog 1 (*EYA1*) and paired box gene 2 (*PAX2*), define the spatial and functional competence of the mesenchyme, directing proper positioning and responsiveness to UB induction signals. Pathogenic variants affecting these regulators are found in both isolated and syndromic forms of CAKUT [32,33,34,35].

### 2.2. UB Expansion and Arborization Programs

Following its emergence, the UB invades the MM and undergoes repeated branching events that eventually form the collecting duct system. This branching is primarily driven by continued GDNF–RET signaling and is modulated by downstream pathways including mitogen-activated protein kinase (MAPK) and phosphoinositide 3-kinase/protein kinase B (PI3K–AKT). Inhibitory signals from molecules such as sprouty homolog 1 (SPRY1) and bone morphogenetic protein 4 (*BMP4*) prevent abnormal or ectopic branching activity [11,14,28]. Branch patterning is additionally shaped by ECM components and signals derived from retinoic acid-responsive stromal cells. Aberrations in these regulatory networks can result in structural abnormalities such as renal hypodysplasia, duplex collecting systems or urinary tract obstructions [1,17,24,36].

### 2.3. Balance of Progenitor Self-Renewal and Nephrogenesis

Nephron progenitor cells must be preserved in an undifferentiated state while also remaining poised for activation. This balance is maintained by transcription factors including *SIX2* and *WT1* and by signaling molecules such as bone morphogenetic protein 7 (*BMP7*) and fibroblast growth factor 20 (*FGF20*). Signals emanating from the ureteric bud, particularly Wnt family member 9B (*WNT9B*), promote mesenchymal-to-epithelial transition through beta-catenin activation, initiating nephron formation. Disruption of this pathway has been implicated in renal hypoplasia and agenesis [9,13,24,37]. Genes involved in ciliogenesis and planar cell polarity, such as those encoding CEPs and oral-facial-digital syndrome-associated proteins, are critical for maintaining tubule orientation and extension. Defects in these mechanisms help explain the link between CAKUT and ciliopathies [1,9].

### 2.4. Spatial Organization of the Ureter and Tubular Architecture

Correct integration of the ureter into the bladder requires input from several signaling pathways, including the *RET* receptor, *BMP4* and the slit guidance ligand/roundabout receptor (SLIT–ROBO) axis. Errors in these pathways can cause VUR, ectopic ureteral insertion or urinary tract obstruction [3,14,37]. Tubulogenesis, the process of tubule formation and elongation, depends on proper apical–basal polarity and basement membrane stability. This structural integrity is mediated by key ECM proteins such as integrins, laminins and type IV collagen. Disruptions in these components can lead to dysplastic kidneys or obstructive phenotypes [17,36].

## 3. Molecular Pathways Underlying CAKUT

CAKUT results from disorders in signaling networks that coordinate UB branching, nephron induction, tubular patterning and urinary tract morphogenesis. These pathways constitute key developmental regulators identified through experimental studies and human genetic analyses [4,5,6,15,25,26]. Recent exome-based analyses further show that perturbations in these pathways often coincide with extrarenal developmental defects, reflecting shared embryologic programs [13].

### 3.1. UB Induction via the GDNF–RET Axis

The GDNF–RET axis is indispensable for UB outgrowth and subsequent branching. GDNF-mediated *RET* activation triggers downstream MAPK, PI3K–AKT and phospholipase C-gamma (PLCγ) pathways, promoting epithelial proliferation and invasion into the MM [10,11,12,13,14,16,27,28,29]. Spatial restriction of UB initiation is achieved through inhibitory inputs from SPRY1, ETS variant transcription factor 4 (ETV4), ETS variant transcription factor 5 (ETV5) and BMP signals derived from surrounding stromal cells, preventing ectopic budding [11,14,28]. Genetic alterations affecting *GDNF*, *RET* or their regulatory components can produce renal agenesis, duplicated systems or obstructive malformations characteristic of CAKUT [30,31,32].

### 3.2. Canonical WNT Signals in Nephron Formation

Canonical WNT/β-catenin signaling leads to nephron induction by enabling UB-derived *WNT9B* to activate β-catenin in nephron progenitors, thereby initiating mesenchymal-to-epithelial transition (MET) and nephron differentiation [9,24,37]. *WNT4* stabilizes epithelial fate and drives tubule formation; disruption produces hypodysplasia or failed nephrogenesis [13]. Within the UB lineage, precise modulation of β-catenin levels ensures orderly branching, and deviation from this balance disrupts collecting duct morphogenesis and contributes to CAKUT-related defects [24]. Mild β-catenin dysregulation has also been linked to variable expressivity in CAKUT, consistent with patient-level observations in sequencing cohorts [14].

### 3.3. Growth Factor Signaling in Branching and Patterning

*BMP4* restricts UB budding to appropriate sites, whereas *BMP7* supports nephron progenitor survival. Disturbance of BMP signaling contributes to renal underdevelopment and ureteral malposition [1,17,36]. *FGF20* and *FGF9* maintain progenitor self-renewal, and extracellular signal-regulated kinase (ERK)-dependent FGF signaling integrates with *RET* activity to refine branching dynamics [14]. SHH, produced by the ureteric epithelium, patterns the surrounding mesenchyme and is essential for ureteral smooth muscle formation and patency. Abnormal SHH signaling leads to ureteral obstruction and hydronephrosis [3].

### 3.4. Matrix Dynamics and Epithelial Integrity

The ECM provides structural scaffolding and transduces mechanical cues required for UB branching and tubular morphogenesis. Collagen IV, laminins, integrins and proteoglycans regulate epithelial polarity and lumen formation, while disruptions in these components lead to dysplastic kidneys and disordered branching [17,36]. Genetic evidence implicating ECM-modifying enzymes and basement membrane components further supports the contribution of adhesion and matrix pathways to CAKUT pathogenesis [24]. Recent exome data also identify ECM-related variants in CAKUT patients, reinforcing the role of matrix integrity in renal morphogenesis [14].

### 3.5. Ciliogenesis and Polarity Cues in Tubular Architecture

Primary cilia coordinate mechanosensation and transduce SHH, WNT and platelet-derived growth factor (PDGF) signals essential for tubular growth and orientation. Mutations in ciliary and centrosomal genes (including several CAKUT-associated loci) impair lumen formation and contribute to CAKUT-related ciliopathies [1,9]. Planar cell polarity pathways regulate directional cell rearrangements and tissue elongation; defects in these mechanisms produce shortened, dilated or obstructed tubules and link PCP dysfunction to urinary tract malformations [9]. Sequencing studies further highlight ciliary and PCP genes as recurrently affected categories in CAKUT cohorts [13]. A summary of the major developmental pathways, their molecular components and representative CAKUT phenotypes is presented in Table 1.

## 4. Genetic Etiology of CAKUT

**Figure 1:**
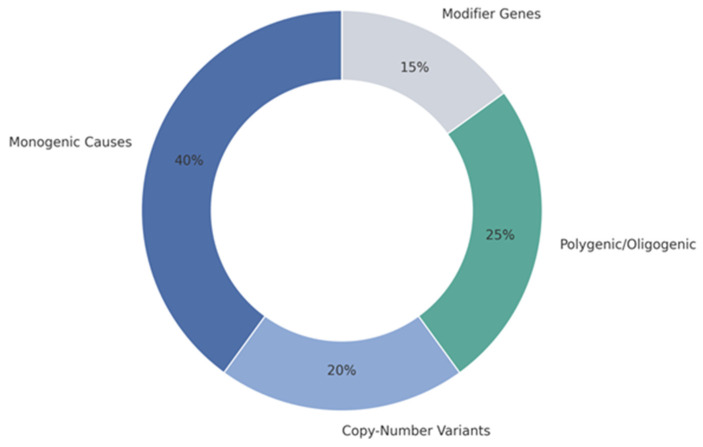
Relative contributions of major genetic mechanisms underlying congenital anomalies of the kidney and urinary tract (CAKUT).

CAKUT arises from diverse genetic mechanisms including monogenic variants, recurrent CNVs and combined polygenic or oligogenic influences (Figure 1).

The schematic illustrates the relative contributions of monogenic variants, copy-number variants and multigenic architectures to CAKUT pathogenesis, highlighting their convergence on key developmental pathways regulating ureteric bud induction, nephron formation and urinary tract morphogenesis.

Developmental programs that coordinate UB branching, nephron formation and urinary tract organization represent key genetic vulnerability points [4,5,6,15,25,26]. Recent sequencing efforts further indicate that many CAKUT-associated variants occur in genes with broader developmental roles, contributing to variable expressivity [13]. The principal genetic categories of CAKUT and representative genes are outlined in Table 2.

### 4.1. Single-Gene Defects in Key Developmental Pathways

Monogenic CAKUT involves genes controlling transcriptional regulation, UB–MM communication, ciliary function and ECM integrity. Transcription factors that define early renal lineage identity, including *PAX2*, *EYA1* and *SIX2*, together with spalt-like transcription factor 1 (*SALL1*) and *HNF1B*, are frequent contributors to CAKUT. Disease-associated variants at these loci commonly manifest as renal hypodysplasia, VUR or multicystic dysplastic kidneys [1,9,13,17,24,36,37]. Genes operating within fundamental developmental signaling pathways, including GDNF–RET, *WNT*, *FGF* and *BMP* are also commonly implicated, with pathogenic variants disturbing branching morphogenesis, nephron induction or ureteral patterning [11,14,16,17,27,28,29]. Ciliopathy-related genes that encode proteins of the intraflagellar transport machinery, centrosomes or planar cell polarity pathways contribute to CAKUT by disrupting tubular geometry and sensory signaling [1,9,13]. Additionally, pathogenic changes in ECM components or adhesion molecules alter basement membrane formation and epithelial stability, leading to dysplastic renal phenotypes [17,36]. Large-scale genomic datasets increasingly support these gene sets as major contributors to both isolated and syndromic CAKUT [13].

### 4.2. Structural Genomic Variants and Dosage Effects

Recurrent CNVs represent a major genetic contributor to CAKUT. CNVs affecting chromosomal regions that encode developmental regulators, including loci containing *HNF1B*, *EYA1/SIX2* and genes involved in ciliary or ECM-associated pathways, which are frequently associated with renal hypodysplasia, duplicated collecting systems or obstructive uropathies [13,24,37]. Their impact often reflects gene-dosage alterations that modify expression of key morphogenetic regulators, and these rearrangements commonly present with syndromic features because of the widespread developmental functions of the affected genes [9,24,37]. Clinical sequencing cohorts also highlight CNVs as a frequent cause of CAKUT with expanded extrarenal manifestations [14].

### 4.3. Combined Genetic Burden and Multigenic Risk

The incomplete penetrance of many monogenic variants and the modest effect sizes of individual loci support a polygenic or oligogenic architecture in a substantial proportion of CAKUT cases [4,5,6,15,25,26]. Combinatorial variation across genes regulating UB branching, progenitor maintenance or ciliary signaling can collectively surpass critical developmental thresholds, producing structural defects not evident from single loci alone. Experimental studies further demonstrate that partially functional, hypomorphic alleles in different pathways can interact to generate CAKUT-like phenotypes, underscoring the importance of quantitative modulation in kidney developmental networks [9,13,36]. Genomic burden analyses likewise show enrichment for multiple rare variants per individual, consistent with multigenic contribution in selected patients [13].

### 4.4. Genetic Modifiers and Phenotypic Variability

CAKUT exhibits marked variability even among individuals carrying the same pathogenic variant. Modifier genes influencing WNT, *RET* or BMP signaling can shift the severity, laterality or presence of malformations, while the broader genetic background dictates cellular responsiveness to environmental or epigenetic stressors [11,14,28,30,31,32]. Variants in transcriptional cofactors, regulators of ciliary organization or enzymes involved in ECM remodeling may modify renal phenotypes, producing a continuum that ranges from isolated VUR to severe renal hypodysplasia. This broad spectrum supports a model in which penetrance and expressivity emerge through layered interactions between primary disease-causing alleles and secondary genetic or developmental modulators [9,13,36]. This modifier framework aligns with recent observations of highly variable expressivity among individuals sharing the same pathogenic variant in sequencing cohorts [13].

## 5. Epigenetic Regulation and Prenatal Environmental Impact

Epigenetic processes and prenatal exposures shape the phenotypic landscape of CAKUT beyond monogenic mutations. Family based studies and multi-omics investigations reveal that modifications in chromatin accessibility, RNA dynamics as well as non-coding regulatory elements influence penetrance, laterality and expressivity in congenital renal anomalies [3,13,15].

### 5.1. Chromatin Structure and Transcriptional Access

Chromatin remodeling emerges as a central influence on renal developmental programs. Analyses of human kidney tissue affected by CAKUT show altered accessibility at key regulatory loci, particularly those tied to ECM assembly and intracellular growth pathways like PI3K–AKT [19]. Several transcriptional regulators essential for kidney organogenesis depend on chromatin configuration for proper spatial activation. Mutations in these loci exemplify how compromised transcriptional control may skew early renal patterning [10,11]. Furthermore, disruption of nuclear RNA processing factors such as SON DNA-binding protein (*SON*) alters precursor mRNA (pre-mRNA) maturation across multiple developmental genes, reinforcing the role of nuclear architecture in nephrogenesis [38].

### 5.2. Non-Coding RNAs and RNA Processing Elements

The role of non-coding RNA species, including miRNAs and long non-coding RNAs (lncRNAs), has become increasingly evident in CAKUT. Structural variants involving miRNA loci like miRNA9-3 (MIR9-3) and miRNA1299 (MIR1299) have been linked to aberrant ureteric and nephron development [18]. Experimental models reveal that miRNAs shape ECM integrity while lncRNAs regulate ciliary genes and nephron fate [17,35]. Additionally, altered RNA maturation pathways, illustrated by SON-related splicing defects, affect transcript processing of several CAKUT-associated genes [38].

### 5.3. Maternal Exposures and Developmental Modifiers

Environmental insults such as maternal diabetes, teratogenic exposure or hypoxia interact with genetic vulnerabilities to exacerbate CAKUT phenotypes [3]. These exposures modulate key signaling pathways, including those guided by vitamin A and D, which influence transcriptional programs active in the UB and nephron progenitors [16]. Such interactions help explain variable expressivity and incomplete penetrance frequently observed across CAKUT families [15]. Consistent with this concept, environmental and epigenetic modifiers are increasingly recognized to interact with core developmental signaling pathways during nephrogenesis. For example, retinoic acid and vitamin D signaling have been shown to modulate WNT/β-catenin and BMP-mediated programs, thereby influencing ureteric bud patterning, nephron progenitor maintenance and susceptibility to CAKUT in genetically predisposed individuals.

## 6. Molecular Lesions Driving Distinct CAKUT Presentations

CAKUT comprises diverse renal and urinary tract malformations that stem from perturbations in common developmental pathways. Although many genetic variants act on similar processes, including UB growth, nephron specification and ciliary function, their effects vary depending on timing, dosage and cellular context. This section links specific molecular alterations to the major clinical CAKUT entities.

### 6.1. Loss of Nephron Induction and Kidney Undergrowth

Renal agenesis and hypodysplasia frequently result from impaired communication between the UB and the MM during early kidney organogenesis. Variants in genes such as *GEN1*, *FOXD2*, *TSHZ3* and the splicing regulator *SON* disrupt these reciprocal signals, leading to reduced nephron number or complete absence of renal tissue [5,6,7,8,9,26,33,34,35,38]. Specifically, *SON* haploinsufficiency interferes with pre-mRNA maturation of multiple genes essential for nephrogenesis, contributing to a broad hypoplastic phenotype [36]. Exome data from human cohorts confirms a significant burden of de novo variants in genes controlling chromatin state, cilia assembly and transcription, emphasizing the multigenic basis of impaired renal growth [9,10,13,27,34].

### 6.2. Tubular Cystogenesis in Developmental Context

Cystic kidney anomalies within the CAKUT spectrum are primarily associated with dysfunctions in primary cilia, epithelial polarity and tubular lumen regulation. Mutations in genes such as *CEP78*, pre-mRNA processing factor 8 (*PRPF8*) and dual-specificity tyrosine-regulated kinase 2 (*DYRK2*) affect RNA splicing or ciliogenesis, promoting cystic tubular dilation during development [9]. Clinical sequencing of CAKUT patients reveals recurrent involvement of cilia-associated genes, often accompanied by additional structural urinary tract anomalies [4,6,15]. Additionally, *HNF1B*, a transcription factor critical for tubulointerstitial organization, has been strongly linked to cystic dysplasia, supporting its broader contribution to tubular epithelial maintenance and metabolic homeostasis [11].

### 6.3. Structural and Functional Obstruction of the Urinary Tract

Congenital obstruction of urinary flow, including ureteropelvic junction and bladder outlet obstruction, arises from disruptions in smooth muscle differentiation, ECM dynamics or UB patterning. Genes involved in mesenchymal–epithelial crosstalk, basement membrane architecture and stromal organization are frequently implicated [3,5,15]. Reverse-phenotyping studies reveal that genes not traditionally associated with obstructive lesions may still cause functional blockage when they affect polarity, matrix composition or bud trajectory [29]. CNV analysis further supports the genetic basis of these phenotypes, highlighting regions linked to smooth muscle development and tissue remodeling [18,22].

### 6.4. Malposition of the Ureter and Reflux Pathophysiology

Malformations of ureter insertion and VUR stem from deviations in UB positioning, ECM signaling and the formation of the ureterovesical junction. Pathogenic variants in genes governing this interface are strongly associated with primary VUR [1,37]. Additional studies have identified co-segregation of VUR with variants in transcription factors, cilia-related genes and regulators of tubular elongation [9,13,14,27,39]. Dosage-sensitive non-coding elements, including MIR9-3 and MIR1299, influence ureteric morphology and smooth muscle organization, thereby modulating susceptibility to reflux [18].

## 7. Integrative Approaches to Early Recognition and Personalized Care in CAKUT

Early evaluation of CAKUT relies on a combination of prenatal imaging, genomic testing and molecular biomarkers to refine diagnosis and risk assessment. Prenatal ultrasonography and fetal magnetic resonance imaging (MRI) define structural anomalies, whereas next-generation sequencing (NGS) improves the detection of monogenic and syndromic cases. Transcriptomic, proteomic and miRNA-based analyses contribute emerging biomarkers of prognostic importance and genetic counseling ensures informed clinical as well as reproductive decision-making.

### 7.1. Prenatal Imaging for Early Structural Assessment

Prenatal ultrasonography is the primary tool for early recognition of CAKUT, allowing evaluation of renal morphology, corticomedullary definition, urinary tract dilation and the presence of cystic alterations [2,30]. Fetal MRI provides additional spatial detail, particularly in complex cystic or ambiguous anatomical presentations and improves prediction of postnatal renal performance [30]. Accurate fetal phenotyping is crucial for distinguishing physiologic transient dilation from structural CAKUT, thereby supporting more precise decisions regarding postnatal evaluation, monitoring intensity and genetic testing pathways [2,30]. Key prenatal imaging patterns and their corresponding postnatal risk implications are summarized in Table 3.

### 7.2. Expanded Genetic Testing and Diagnostic Performance of NGS

NGS has become central to the diagnostic approach for CAKUT, particularly through exome sequencing and targeted renal–urinary gene panels. Reported diagnostic yields vary substantially, ranging from approximately 6% in mild or unselected cohorts to nearly 40% in severe or syndromic cases, reflecting differences in phenotypic breadth, age at evaluation and study methodology [1,9,14,27,29,39,40,41]. Trio-based exome sequencing further increases diagnostic yield by identifying pathogenic de novo variants and enabling recognition of novel candidate genes, especially in early-onset or multisystem presentations [9,13,14,27,42]. These strategies also facilitate reverse phenotyping, refining genotype–phenotype interpretation and improving classification of atypical clinical presentations [14,29]. Today, NGS is routinely applied across presentations such as renal agenesis, cystic anomalies, reflux-associated disease and syndromic CAKUT, establishing it as a central component of contemporary clinical genomics [1,27,33,39].

### 7.3. Biomarkers from Integrative Multi-Omics Strategies

Multi-omics investigations have mapped a wide range of pathways relevant to CAKUT pathobiology, highlighting alterations in collagen network integrity and PI3K–Akt signaling [19], defects in ciliogenesis [15] and transcriptional disturbances involving *FOXD2* and *HNF1B* [10,11]. Representative multi-omics platforms and their biomarker outputs relevant to CAKUT are presented in Table 4.

Beyond isolated gene findings, these datasets reveal coordinated changes across transcriptomic, proteomic and epigenomic layers, providing a more integrated perspective on disrupted developmental programs. Developmental-stage transcriptomic profiling demonstrates dynamic shifts in nephrogenic trajectories, reflecting how the timing of pathway disruption shapes phenotype [20,32]. While copy-number variant and miRNA analyses have identified additional regulatory influences, including MIR9-3 and MIR1299 [17,18], their clinical utility increasingly lies in their contribution to composite molecular signatures that distinguish severe from mild disease. Integrated multi-omics platforms therefore provide a foundation for identifying biomarker combinations that may support earlier diagnosis, refined risk prediction and the future development of mechanism-based therapeutic strategies [19,21].

### 7.4. Genetic Counseling Considerations in Clinical Care

Genetic counseling is a crucial component of CAKUT management because of the heterogeneous inheritance patterns, variable expressivity and incomplete penetrance characteristic of both monogenic cases and CNV-associated forms [1,3,5,15,25]. Counselors must address recurrence risks, reproductive planning, prenatal diagnostic options and potential links to multisystem involvement [24,30]. Identification of pathogenic variants allows individualized counseling, supports targeted surveillance for extrarenal manifestations and helps clinicians anticipate long-term renal outcomes [1,3,24,25].

## 8. Research Frontiers and Translational Opportunities in CAKUT

Rapid advances in developmental biology and genomic technology are transforming the landscape of CAKUT research. High-resolution molecular profiling, in vitro modeling and functional genomic tools are now being integrated to address unresolved disease mechanisms, improve variant interpretation and inform precision medicine strategies. Emerging platforms such as spatial transcriptomics, kidney organoids and clustered regularly interspaced short palindromic repeats (CRISPR)-based screening contribute to a deeper understanding of renal morphogenesis and support the development of targeted, mechanism-based interventions for CAKUT. Recent exome-based analyses have further expanded the developmental pathways implicated in CAKUT and refined gene prioritization for functional follow-up [13].

### 8.1. High-Resolution Transcriptomic Platforms in Renal Development

Single-cell and spatial transcriptomic technologies enable detailed mapping of nephrogenesis by revealing cell-type specific expression profiles and developmental anomalies. Abnormal localization of transcripts related to CAKUT, including discs large homolog 1 (DLG1) and kinesin family member 12 (KIF12), has been observed in human fetal kidney tissue [20,32]. These platforms enhance gene prioritization and aid in elucidating previously unrecognized mechanisms in genetically unexplained cases, complementing insights from developmental exome studies [13,21].

### 8.2. In Vitro Organoid Systems for Modeling Renal Morphogenesis

Kidney organoids derived from human pluripotent stem cells recapitulate key stages of renal development and serve as functional models to investigate the effects of pathogenic variants, including those affecting *FOXD2* and *HNF1B* [10,11]. Integration with transcriptomic and proteomic analyses allows for exploration of developmental timing, tissue-specific phenotypes and molecular pathways relevant to CAKUT [21].

### 8.3. Gene Perturbation Tools and Functional Validation Strategies

CRISPR-based technologies provide efficient platforms for functional testing of candidate genes identified in sequencing studies. Variants in genes such as *PRPF8*, *DYRK2* and *CEP78* have been investigated using genome editing to evaluate their contribution to renal morphogenesis [9,13,42]. These tools facilitate interpretation of variants of uncertain significance and improve mechanistic clarity within disease-related gene networks, aligning with observations from clinical exome sequencing studies that emphasize the need for functional validation of candidate variants [9,14,15].

### 8.4. Developmental Signaling as a Target for Precision Therapies

Targeting key developmental signaling pathways represents a promising therapeutic direction in CAKUT. Aberrations in the PI3K–Akt axis, ECM remodeling and nutrient-responsive transcriptional programs have been implicated in multiple CAKUT subtypes [16,19]. Mechanistic insights into UB dynamics and ciliary function continue to guide the identification of molecular targets with potential for future precision-based interventions [5,15,21].

Despite major advances in developmental biology and genomic technologies, important gaps persist in the current understanding of CAKUT pathogenesis. A substantial proportion of patients still lack an identifiable genetic diagnosis, highlighting limitations in variant interpretation, particularly for noncoding regions, structural variants and complex multigenic architectures. Genotype–phenotype correlations remain incomplete, with marked variability even among individuals carrying identical pathogenic variants. Moreover, experimental and in vitro models, although highly informative, do not fully recapitulate the spatial, temporal and environmental complexity of human nephrogenesis. Addressing these challenges will require integrative strategies combining high-resolution genomics, human tissue-based studies and longitudinal clinical phenotyping.

## 9. Conclusions

The CAKUT spectrum includes a wide range of malformations that arise from disruptions in critical developmental processes such as UB induction, branching morphogenesis, nephron differentiation and urinary tract organization. These anomalies reflect perturbations in transcriptional control, signaling networks, ciliary dynamics and ECM architecture. Genetic contributions include monogenic mutations, recurrent copy-number variants and complex polygenic or oligogenic architectures, while epigenetic mechanisms in combination with environmental exposures further modulate expressivity and penetrance.

Recent advances in high-throughput sequencing and multi-omics profiling have substantially improved the understanding of CAKUT etiology, revealing both known and novel disease genes as well as mechanistic pathways. Integration of prenatal imaging with genomic diagnostics enables earlier recognition, while transcriptomic and functional data refine variant interpretation and illuminate pathogenesis in genetically unsolved cases. Genetic counseling has become a critical tool for risk assessment, familial management and anticipation of syndromic involvement.

Future directions in CAKUT research include the application of single-cell and spatial transcriptomics, refinement of kidney organoid platforms and implementation of CRISPR-based functional screens. These emerging technologies are expected to narrow the gap between genotype and clinical phenotype, particularly in genetically unresolved cases, and to improve interpretation of noncoding, structural and multigenic variation. Continued integration of developmental biology, human tissue-based studies and genomics will be essential for overcoming current limitations, informing the rational design of targeted therapies and enabling a more individualized approach to patient management in congenital renal anomalies.

## Figures and Tables

**Table 1:** Developmental pathways and representative CAKUT phenotypes. This table summarizes key embryologic signaling pathways involved in kidney and urinary tract development, highlighting their principal molecular components, mechanisms of disruption and the resulting CAKUT phenotypes. By linking developmental processes with molecular perturbations and clinical manifestations, the table provides an integrated framework for understanding pathway–phenotype relationships in CAKUT.

Developmental Pathway	Key Molecular Components	Mechanistic Disruption	Representative CAKUT Phenotypes
GDNF–RET induction	*GDNF*, *RET*, GFRα1	Impaired UB outgrowth	Renal agenesis, duplex kidney
WNT/β-catenin signaling	*WNT9B*, *WNT4*, β-catenin	Defective MET and nephron induction	Renal hypoplasia, dysplasia
BMP/FGF axis	*BMP4*, *BMP7*, *FGF9/20*	Abnormal bud positioning, failed progenitor maintenance	Hypodysplasia, ureteral malposition
ECM and polarity	Laminins, integrins, collagen IV	Loss of apical–basal polarity	Dysplastic kidneys
Ciliogenesis and PCP	IFT proteins, *CEP78*, *DYRK2*	Abnormal lumen formation and orientation	Cystic anomalies, obstruction

UB, ureteric bud; *GDNF*, glial cell line-derived neurotrophic factor; *RET*, rearranged during transfection; GFRα1, *GDNF* family receptor alpha-1; WNT, Wingless/Integrated; MET, mesenchymal-to-epithelial transition; BMP, bone morphogenetic protein; FGF, fibroblast growth factor; ECM, extracellular matrix; PCP, planar cell polarity; IFT, intraflagellar transport; *CEP78*, centrosomal protein 78; *DYRK2*, dual-specificity tyrosine-regulated kinase 2.

**Table 2:** Genetic categories of CAKUT with representative genes and mechanisms. This table summarizes the major genetic architectures underlying CAKUT, including monogenic, copy-number variant and oligogenic models, highlighting representative genes, shared mechanistic themes and modifiers of disease expression. By integrating genetic category with molecular mechanism and phenotypic variability, the table provides a conceptual framework for understanding incomplete penetrance, variable expressivity and syndromic presentations in CAKUT.

Genetic Category	Representative Genes	Mechanistic Theme	Notes
Monogenic	*HNF1B*, *PAX2*, *SALL1*, *SIX2*	Transcriptional and developmental control	Variable expressivity, renal underdevelopment
CNVs	17q12 (*HNF1B*), 10q21.1 (*EYA1/SIX1*), miRNA loci	Dosage effects on pathways	Syndromic presentations common
Oligogenic	*RET* + *GDNF* + modifiers	Threshold-dependent developmental disruption	Explains incomplete penetrance
Modifier genes	ECM remodelers, ciliary regulators	Tune severity and laterality	Influence phenotype within families

CNV, copy-number variant; *HNF1B*, hepatocyte nuclear factor 1-beta; *PAX2*, paired box gene 2; *SALL1*, spalt-like transcription factor 1; *SIX2*, sine oculis homeobox homolog 2; *EYA1*, eyes absent homolog 1; *SIX1*, sine oculis homeobox homolog 1; miRNA, microRNA; *RET*, rearranged during transfection; *GDNF*, glial cell line-derived neurotrophic factor; ECM, extracellular matrix.

**Table 3:** Prenatal imaging findings and corresponding risk implications. This table summarizes common prenatal ultrasound and imaging findings associated with CAKUT and links them to postnatal risk stratification and recommended follow-up strategies. By integrating imaging phenotypes with clinical risk and management considerations, the table provides a practical framework for early counseling and postnatal evaluation.

Prenatal Finding	Interpretation	Postnatal Risk	Suggested Follow-Up
Mild pyelectasis	Possible physiologic dilation	Low	Postnatal US
Bilateral hydronephrosis	Obstruction or reflux	Moderate–high	Early nephrology/Urology referral
Cortical thinning	Parenchymal damage	High	Full work-up, consider NGS
Cystic lesions	Cystic dysplasia/syndromic CAKUT	High	Genetics + MRI + serial monitoring
Absent kidney	Agenesis	High	Full CAKUT screen and extrarenal evaluation

MRI, magnetic resonance imaging.

**Table 4:** Multi-omics modalities and their biomarker outputs.

Omics Platform	Measurement	Example Markers	Clinical Relevance
Transcriptomics	Gene expression	*FOXD2*, ECM signatures	Distinguishes severity and timing of disruption
Proteomics	Secreted proteins	ECM remodeling enzymes	Potential urine biomarkers
miRNA profiling	Regulatory non-coding RNAs	MIR9-3, MIR1299	Links CNVs to phenotype
Epigenomics	Chromatin and methylation	Loci affecting cilia/ECM	Explains variable penetrance
Integration (multi-omics)	Combined signatures	Composite developmental modules	Precision diagnosis and prognosis

*FOXD2*, forkhead box D2; ECM, extracellular matrix; miRNA, microRNA; MIR9-3, microRNA 9-3; MIR1299, microRNA 1299; CNVs, copy-number variants.

## Data Availability

The data supporting the reported results are from previously published data presented in the study are openly available in all the academic databases mentioned in the method section.

## Abbreviations

The following abbreviations are used in this manuscript:

CAKUT	Congenital Anomalies of the Kidney and Urinary Tract
UB	Ureteric Bud
MM	Metanephric Mesenchyme
*GDNF*	Glial Cell Line-Derived Neurotrophic Factor
*RET*	Rearranged During Transfection (receptor tyrosine kinase)
GFRα1	*GDNF* Family Receptor Alpha-1
*WT1*	Wilms Tumor Protein 1
*SIX2*	Sine Oculis Homeobox Homolog 2
SIX1	Sine Oculis Homeobox Homolog 1
EYA1	Eyes Absent Homolog 1
*PAX2*	Paired Box Gene 2
SALL1	Spalt-Like Transcription Factor 1
*HNF1B*	Hepatocyte Nuclear Factor 1-Beta
BMP	Bone Morphogenetic Protein
*BMP4*	Bone Morphogenetic Protein 4
*BMP7*	Bone Morphogenetic Protein 7
FGF	Fibroblast Growth Factor
*FGF20*	Fibroblast Growth Factor 20
*FGF9*	Fibroblast Growth Factor 9
MAPK	Mitogen-Activated Protein Kinase
PI3K	Phosphoinositide 3-Kinase
AKT	Protein Kinase B
PLCγ	Phospholipase C-Gamma
SPRY1	Sprouty Homolog 1
ECM	Extracellular Matrix
MET	Mesenchymal-to-Epithelial Transition
WNT	Wingless/Integrated Signaling Family
*WNT9B*	Wingless/Integrated Protein 9B
SHH	Sonic Hedgehog
DLG1	Discs Large Homolog 1
KIF12	Kinesin Family Member 12
*PRPF8*	Pre-mRNA Processing Factor 8
CEP78	Centrosomal Protein 78
*DYRK2*	Dual-Specificity Tyrosine-Phosphorylation-Regulated Kinase 2
PCP	Planar Cell Polarity
PDGF	Platelet-Derived Growth Factor
miRNA	MicroRNA
lncRNA	Long Non-Coding RNA
MIR9-3	MicroRNA 9-3
MIR1299	MicroRNA 1299
CNV	Copy-Number Variant
NGS	Next-Generation Sequencing
ES	Exome Sequencing
WES	Whole-Exome Sequencing
WGS	Whole-Genome Sequencing
MRI	Magnetic Resonance Imaging
VUR	Vesicoureteral Reflux
